# Intimate partner violence in the post-war context: Women’s experiences and community leaders’ perceptions in the Eastern Province of Sri Lanka

**DOI:** 10.1371/journal.pone.0174801

**Published:** 2017-03-31

**Authors:** Sepali Guruge, Marilyn Ford-Gilboe, Colleen Varcoe, Vathsala Jayasuriya-Illesinghe, Mahesan Ganesan, Sivagurunathan Sivayogan, Parvathy Kanthasamy, Pushparani Shanmugalingam, Hemamala Vithanarachchi

**Affiliations:** 1 Daphne Cockwell School of Nursing, Ryerson University, Toronto, Ontario, Canada; 2 Schulich School of Medicine and Dentistry, Western University, London, Ontario, Canada; 3 School of Nursing, University of British Columbia, Vancouver, British Columbia, Canada; 4 National Institute of Mental Health, Colombo, Sri Lanka; 5 Faculty of Medical Sciences, University of Sri Jayewardenepura, Sri Lanka; 6 Vasantham, Toronto, Canada; 7 University of Colombo, Colombo, Sri Lanka; 8 Open University, Colombo, Sri Lanka; Stellenbosch University, SOUTH AFRICA

## Abstract

**Background:**

Exposure to armed conflict and/or war have been linked to an increase in intimate partner violence (IPV) against women. A substantial body of work has focused on non-partner rape and sexual violence in war and post-war contexts, but research about IPV is limited, particularly in Asian settings. This paper presents the finding of a study conducted in the Eastern Province of Sri Lanka. The study explored women’s experiences of and responses to IPV as well as how health and social service providers perceive the problem. It also explored the IPV-related services and supports available after the end of a 30-year civil war.

**Method:**

We conducted in-depth, qualitative interviews with 15 women who had experienced IPV and 15 service providers who were knowledgeable about IPV in the Eastern Province of Sri Lanka. Interviews were translated into English, coded and organized using NVivo8, and analyzed using inductive thematic analysis.

**Results:**

Participants described IPV as a widespread but hidden problem. Women had experienced various forms of abusive and controlling behaviours, some of which reflect the reality of living in the post-war context. The psychological effects of IPV were common, but were often attributed to war-related trauma. Some men used violence to control women and to reinstate power when their gender roles were reversed or challenged due to war and post-war changes in livelihoods. While some service providers perceived an increase in awareness about IPV and more services to address it, this was discordant with women’s fears, feelings of oppression, and perception of a lack of redress from IPV within a highly militarized and ethnically-polarized society. Most women did not consider leaving an abusive relationship to be an option, due to realistic fears about their vulnerability to community violence, the widespread social norms that would cast them as outsiders, and the limited availability of related services and supports.

**Implications:**

These findings revealed the need for more research about IPV in post-war contexts. Women’s experiences in such contexts are influenced and may be masked by a complex set of factors that intersect to produce IPV and entrap women in violence. A more nuanced understanding of the context-specific issues that shape women’s experiences of IPV- and community responses to it—is needed to develop more comprehensive solutions that are relevant to the local context.

## Introduction

Intimate partner violence (IPV) refers to behaviours by a current or former intimate partner that cause physical, sexual or psychological harm, including physical aggression, sexual coercion, psychological abuse, and controlling behaviours [1]. Globally, women bear the greatest burden from IPV, which is known to be a complex human rights violation rooted in gender and structural inequalities within a society [2,3]. Research focusing on the nature and effects of IPV and interventions to prevent or reduce it has grown exponentially, but significant gaps remain in understanding IPV and other forms of gender-based violence in war and post-war contexts, particularly in low- or middle-income countries [4]. Exposure to violence during armed conflict/war has been linked with other forms of gender-based violence against women [5,6], but most research has explored non-partner rape and sexual assault in these contexts [7]. A few studies have reported increased IPV during periods of armed conflict in countries including Lebanon, the Palestinian territories [8,9], Afghanistan [10], and Sri Lanka [10].

Sri Lanka, the setting for this study, is a South Asian island nation that is emerging from a 30-year long civil war. The war had strong ethnic and geographical dimensions: it was driven by demands from a separatist, predominantly Tamil, ethnic minority group in the northern and eastern regions of the country for a state independent from the Sinhalese majority. The war ended in 2009, but in 2015 more than 70,000 persons were still displaced within war-affected areas [11]. There have also been reports of continued violence, arbitrary arrests, disappearances, and killings in these regions even after the end of the war [12]. Gender-based violence against women has also been reported in these areas [13], but less is known about IPV. A few studies have reported that IPV was common in the northern and eastern regions during and after the war, and that it is more common than non-partner violence [10,14], but details about IPV in the post-war context are not well documented. This paper reports the findings of a study focusing on IPV against women in the Eastern Province of Sri Lanka in the post-war context.

## Background

### The Sri Lankan context

Sri Lanka has a population of approximately 20 million [15] consisting of an ethno-religious mix of Sinhalese (75%), Tamils (11%), Moors (Muslims) (9%), and other groups such as Burgers and Malays (5%). Administratively, Sri Lanka is divided into 9 provinces, and 3 economic sectors: urban, rural, and estate (tea plantations). Most people live in rural areas (77%), and there is a regional segregation by ethnic-religious groups. For example, Tamil Hindus predominate in the Northern and Eastern Provinces, and Sinhalese Buddhists predominate in the rest of the country. Moors (Muslims) have a significant presence in the Eastern Province, but are scattered throughout the country. This study was conducted in the Eastern Province, which is divided into three administrative districts: Ampara, Batticaloa, and Trincomalee. According to the 2012 Census of Sri Lanka [15], the population of Eastern Province consisted mostly of Tamils (40%) and Moors (40%).

Some economic and infrastructure development efforts have been made in the war-affected areas since end of the war in 2009, but recovery and reconciliation efforts have been slow and divisive political agendas continue to promulgate ethnic and political tensions across the country [12]. Approximately 15% of the country’s population live below the poverty line [16], and the people in the war-affected Northern and Eastern Provinces are in the worst circumstances. For example, in 2012 the number of maternal deaths, an important indicator of population health, was much higher in the Northern Province (110 deaths per 100,000 live childbirths) compared to the national average (22 deaths per 100,000 live childbirths). Rates in the Eastern Province were also higher than the national average, ranging from 52–70 deaths per 100,000 live childbirths [17].

### IPV in the Sri Lankan context

A scoping review of studies published about IPV in Sri Lanka [18] revealed that 20–60% women in different parts of Sri Lanka reported experiencing IPV at some point in their life. National prevalence rates are not available, and women tend to under-report experiences of IPV, so actual IPV rates are likely much higher. Most research about IPV in Sri Lanka has focused on Sinhalese majority communities, particularly during the war. The few studies that have included the war-affected Northern and Eastern Provinces and thus included the minority Tamil and Muslim ethnic communities have reported that IPV is common [19] but under-reported by women [20]. A diverse set of correlates that influence women’s vulnerability to IPV have been documented across Sri Lanka, including victim characteristics such as young age [21,22], low socioeconomic status [23,24], low educational attainment [25], as well as abuser characteristics such as sexual jealousy, relationships with multiple sexual partners [23,24,25,26,27], and childhood experiences of violence [27]. Men’s alcohol use/dependence has also been associated with perpetration of IPV alone and/or in combination with other factors such as concomitant drug abuse and low socio-economic status [24,25,28,29]. As in other settings where IPV is pervasive, many of these risk factors appear to be grounded in patriarchy-based cultural, religious, and societal attitudes about gender roles and expectations [30]. Emerging evidence suggests that these factors could interact with other contextual factors in the post-war context to make the situation worse for some women or some groups of women [8]. However, this phenomenon has not been explored in the Eastern Province of Sri Lanka. Therefore, this study explored women’s experiences of and responses to IPV as well as service providers’ perspectives about the nature of the problem of IPV; it also explored the related services and supports available to women within the post-conflict context of the Eastern Province of Sri Lanka.

## Methods

After obtaining ethics approval from all participating universities with which the investigators were affiliated, data collection began with a brief document analysis and informal interviews with community leaders who were knowledgeable about the problem, service providers with experience working in this area, and a few women who and have had experienced IPV. The information gathered from these sources was used to develop interview guides for formal in-depth interviews with health and social service providers and women who had experienced IPV.

### Interviews with health and social service providers

With the help of our existing contacts and networks, we recruited a diverse sample of service providers including nurses, doctors, and midwives working in government hospitals and community clinics; social workers working with governmental and non-governmental organizations; and academics and government agents within the region. These individuals were chosen because of their close connections to the community and their knowledge and experiences working in this field. After obtaining written consent, each participant was interviewed privately by a trained research assistant (RA) at a local community center or clinic; some chose a room in their own place of work. Interviews were offered and conducted in either English or Tamil according to each participant’s preference. Each interview lasted 1–2 hours and was audio-recorded with the participant’s written consent. Participants were informed verbally and via the consent form of their right to refuse to participate or answer any specific questions, and to end the interview at any time. The initial interviews provided useful insights into the local context and helped modify the interview guide used for subsequent interviews. Participants spoke about IPV, the various conditions that influence women’s vulnerability to IPV, and the availability, allocation, and use of services/support in this region. Overall, the interviews helped capture the broad geo-political, social, and cultural contexts, which could not be obtained only from the accounts of individual women who had experienced IPV. Participants were also asked to give feedback on the preliminary interview guide, particularly, the social and cultural appropriateness of the questions included.

### Interviews with women who had experienced IPV

After completing the initial interviews, we used our community contacts and networks, as well as snowball sampling, to recruit 15 women from the Eastern Province who had experienced IPV. Those who were interested in participating contacted a female RA to obtain more information and to arrange interviews. Interviews were conducted at a convenient location and were audio-recorded after obtaining written consent. The written consent form was provided in the language of their choice and contained information about the study, what participants would be asked to do, their right to refuse to participate or answer any specific questions, and their right to end the interview at any time. Most interviews were conducted at a local clinic or a community center. They were offered in all 3 languages spoken in Sri Lanka (Sinhalese, Tamil, and English); with the exception of two participants, all interviewees chose Tamil. Each interview took 1–2 hours and was audio-recorded. An honorarium was provided to all the participants to help defray the costs of participation (i.e. time, transportation, and/or childcare).

Participants were provided with information about local agencies providing services for women experiencing IPV, if they thought it was safe to keep such information with them. The interviewer also paid attention to verbal and non-verbal cues to anticipate any signs of undue discomfort or stress; if she observed any signs, she checked with the participant to see whether she wanted to postpone or end the interview. No participants ended an interview prematurely or withdrew from the study.

Both sets of interview recordings (service providers and women who had experienced IPV) were transcribed verbatim, and interviews conducted in Tamil were translated into English by the RA who conducted the interviews. The translations were randomly checked by another RA who was fluent in Tamil. Next, the two sets of interview data were analyzed separately. The team members read the first three transcripts individually and then met to discuss the codes and to develop consensus on the initial coding scheme. This scheme was then used to code the remaining transcripts using NVivo 8. New codes were compared with previously developed codes to identify commonalities and variations, and to develop subcategories and categories. Common and divergent themes were also identified within and across the two sets of data. To ensure trustworthiness, we used data triangulation (field notes and interviews with women as well as service providers), peer debriefing and review (with other local researchers from the field), and member checks with participants (i.e., each participant during her interview and with other participants in subsequent interviews).

## Results

Health and social service providers included three doctors, three nurses, and three midwives, two social workers (one from the ministry of social services and the other from a local NGO), and one district-level director of health services. This group also included two researchers focusing on violence against women, and one women’s rights advocate who was well-known in the local community. Most participants in this group were Tamil, and middle-aged (40–55 years), eight were women. Almost all chose to be interviewed in English.

The 15 women who had experienced IPV ranged between18–55 years, and represented the ethnic groups in the Eastern Province: eight were Tamil, five were Moor, and two were Sinhalese. All were/had been married to abusive partners, and the majority (11) were still-living with them at the time of the interview. Of the four women who were separated from their abusive partners, two were remarried and two were living with their extended family.

The next section presents the findings from the thematic analyses of the interviews, comparing service providers’ perceptions with women’s experiences of IPV, its consequences, and their responses. Each participant is identified by a number (1–30) preceded by the letters *SP* for health and social provider, *AA* for academics/advocate, and *W* for women with experiences of IPV. The data gathered through initial informal interviews and field work provided the contextual information that was needed to develop the themes as well as to connect the findings to the unique local context. It was incorporated into the development of the themes and the discussion. We examined commonalities and differences within and between statements by service providers and women to explore everyday assumptions, agendas, and power relations in the post-conflict context of the Eastern Province of Sri Lanka. Specifically, we examined perceptions about IPV and the responses of women and their communities to IPV in the context of shifting gender and power dynamics between men and women.

### The problem of IPV: Widespread, yet hidden

The service providers and academic talked about IPV in the Eastern province as a widespread, but hidden problem. They perceived that all forms of IPV was prevalent in the Eastern Province, but some types of abuse such as psychological abuse, was less visible than other forms like physical abuse.

I think it [IPV] is very common [here]. It’s one of the most underreported issues, one of the most hidden issues which nobody wants to talk about. But we all know it happens much more than it is being reported or talked about. So, I think it is widely prevalent.

(AA 12)

We can’t see psychological abuse. Physical abuse we can see and treat. I think the percentage of psychological abuse and harassment is much higher.

(SP 15)

The women interviewed spoke about their personal experiences of various forms of abuse, including physical, sexual, and verbal abuse, and controlling behaviours. Majority had experienced more than one form of abuse; most commonly, a combination of physical and sexual violence. Similar to what was noted by the service providers said, women described various forms of emotional or psychological abuse, including neglect, belittling, and humiliation.

He hit me a lot. He burnt me with an iron rod, stabbed me with a knife. He broke my nose and kicked my lip. After my son was born, the very next day he poured hot water on me. He used to hit me with whatever he gets his hands on such as sticks, a coconut scraper.

(W 24)

He bit me all over my body. He used to pull my dress and tear it. Most of the times he would force me to have sex with him and he hits me a lot and I could not bear it.

(W 22)

He never gave us food; we end up living without food for days. Most of the time he tortured me to sell the house; he regularly asks me to sign on a blank paper so he can sell my house. I got cheated by him many times. He likes to put me to shame.

(W 27)

### Ethnic dimensions of IPV experience in the post-war context

Service providers talked about IPV as being differently experienced by the Tamil, Muslim, and Sinhalese women in the Eastern province. While some believed the problem to be most severe for the Tamil women, others considered Muslim women to be at highest risk. Everyone perceived that Sinhalese women were less affected when compared to Tamil and Muslim women.

Tamil women have got affected more than Sinhalese women. If you take the Muslim community, they are most affected than the other two communities.

(SP 05)

Some of the risks were associated with the different laws that applied to the ethnic groups, for example, the Muslim women’s experiences were shaped by their own communities’ rules and justice procedures, which are believed to be more restrictive than the general marriage/divorce laws in Sri Lanka. Some stated that this was a reason why Muslim women were more likely to becoming entrapped in abusive relationships than Tamil or Sinhalese women.

Usually Tamil women can easily get the divorce; they can apply and get it. Because she is a Muslim, they have a lot of procedures like Cardi court [which makes it difficult].

(SP13)

Others identified risks associated with ethnicity: simply being a Tamil woman was identified as a reason for increased vulnerability to IPV in the post war context.

I think among the Tamil community the percentage [experiencing IPV] can be very high compared to Sinhalese community.

(SP 17)

Most service providers attributed this to the community’s frequent exposure to violence during the war, and that they may have developed a higher tolerance to violence, seeing it as the only option to dealing with conflict. One social worker said: *“People who are used to seeing violence can become violent themselves*. *They don’t have an alternative way to let it out (SP 14)*.*”*

They also talked about the IPV being experienced by under-age girls who are being given in marriage in the region. One social worker who believed this was a reason for increase in IPV said it was common for 15-year-old Muslim and Tamil girls to get married in this area. He said: *“Under-age marriages are taking place among 15 years old children*. *In XXX*, *Divisional Secretarial most of them got married under-aged (SP 08)*.*”*

Overall, IPV was seen as being experienced disproportionately by Tamil and Muslim ethnic groups both as a result and response to war related trauma and distress in the region, and also because of the inherent risks associated with the restrictions imposed by Muslim laws.

### Coercive control and entrapment in abusive relationships

Service providers and women spoke about extreme control of women by men as part of the abusive experience. For some women, this meant not being allowed to leave the house alone, talk to other men, or go out to work. One midwife said: “*This man used to keep his wife inside the house*, *when he goes to work*, *he locks the door and sweeps the garden neatly*, *so that [he will know] if someone comes to the house [because] they will leave foot prints [in the sand] (SP 12)*.*”*

According to service providers, the men prevented women from going out alone in order to stop them from meeting other men. According to women, men were concerned when women went out alone, because of men’s suspicions about women being or becoming sexually promiscuous. One woman said: “*He doesn’t like me to speak with any men*, *he always suspects me*. *He thinks that I might go [have sex] with other men (W 17)*.*”*

Another woman said: *“If I got a little late [to return from the hospital] he used to think that I was having something [an affair] with the doctors*. *He used to make up stories like he saw that*. *He would fight with me entire nights saying that I was involved with this man (W 18)*.*”*

Interestingly, while talking about the men’s control of their lives and the abuse inflicted by them, some women also referred fears of being ‘chased away from home,’ revealing their dependency on their abusive partners. For example, one woman said: “*He always says he will chase me out of that house*. *I ask him if you chase me out of this house where I can go*? *(W 29)*.*”* Others referred to their partners threatening to chase them away from home if they stepped out, talked to other men, and/or were suspected of behaving promiscuously.

### Violence as a means of re-exerting control and power

Some service providers considered IPV to be directly linked to post-war changes in men’s and women’s lives. After the war, many men lost their livelihoods or could not find work, while women gained more access to work outside the home. As a result, women are perceived to have more opportunities to meet or talk to other men. One social worker said: “W*hen the women get jobs outside the home they get looked at in different ways’ (SP 08)*,*” i*.*e*. as women of ‘loose morals.’

The reversal of gender roles and diminution of men’s role in the family were seen as contributing to conflict within the family and violence against women.

The root cause [of IPV] are various things related to masculinity and not being able to fulfil your role because of the conflict and not being able to work or contribute in the family. The insecurity and the fear enables that frustration and the anger.

(SP 09)

In conflict affected areas a lot of women [are] heads of households. Gender roles are changing now, women are going out and earning an income which challenges the notions of masculinity among men which can also trigger conflict and violence within families.

(AA 12)

This was seen as a significant problem to the extent that one social worker recommended self-employment for women, so that they can stay home and avoid going out and being seen as promiscuous, not only by their partners, but within the wider community: “*If we get them self-employment we are able to bring them to a good standing in the community* (*SP 08)*.*”*

The women also said that men often used violence as a means of re-exerting control over them and preventing them from going out:

He suspects me all the time, he will make up a big story. Even if I talk to a woman he thinks that they [the other women] have boyfriends.

(W 25)

He always suspects me, with other men. If I go to the town, he thinks I am having sex with my first husband. He used to hit me saying I am sleeping with my ex-husband.

(W 24)

### Poverty, alcoholism, and men’s extra marital affairs

Service providers said that men’s alcohol use is related to both poverty and IPV, and noted that almost all the men in the region had alcohol-related problems.

This happens in our district because of alcoholism. Because of the consumption of alcohol, the [household] economy totally gets affected. This leads to quarrels among them, and this is a reason for the violence.

(SP 15)

Majority of the men [in the area] are addicted to alcohol, so they spend all of their income on this.

(AA 03)

Poverty and alcoholism were seen as interconnected parts of a complex problem related to lack of jobs for men, spending money on alcohol, combined with relationship problems.

Among the community most of the time problems create because of poor economy. When speaking about economic problem that means men goes for labor work, women will be house wives and they have 6–8 children. Husbands gives them only 10% from their total income. The 10% is not sufficient for food for these 6, 7 children, they expect the wives to prepare a good meal to eat when they come home after having alcohol. If she doesn’t give a proper meal, he abuses his wife and asks [her]to borrow money from others or asking her to go to work.

(AA 04)

Women also considered men’s alcohol use to contribute to IPV, either directly or indirectly though resultant poverty.

When he comes home after drinks, he creates a problem, and makes it a big issue and then starts hitting me.

(W 16)

95% of men [in the area] are addicted to liquor, so they spend the 90% of their income for liquor and 10% for food.

(W20)

Service providers and women talked about men having, or having had, extra-marital sexual relations as a reason for relationship problems and abuse. One woman said: “*He had lot of girlfriends*. *I don’t like this*. *Because of this we had problems regularly*. *Recently he had an affair with a young woman*, *this is not good you know*, *the community talks about these things*, *you know*, *all these things are very bad (W 21)*.*”*

Another woman said: “*He had an affair with another woman I even saw condoms in his purse*. *When I asked him he started to fight with me*? *He had extra marital relationship with his brother’s wife (W 29)*.*”*

### The masking of consequences of IPV

Service providers spoke about the effects of IPV they had observed while working with women who had experienced IPV, which were similar to those described by women: physical injuries including bruises, wounds, lumps, burns, broken arms, and black eyes. In addition to these, they also referred to health problems such as headaches, vomiting, fainting, high blood pressure, and feeling tired all the time.

He used to hit my hands and I got wounded. I have [high] blood pressure after he started to hit me. Once he hit me. My eye got affected and I had a lump too, so I went to get treatment for this.

(W 20)

I have wounds all over the body, and he broke my arm too. Burnt me with fire wood, can you see the marks? I have wounds and marks all over my body. I don’t see at least one part of my body without a wound. I get bad headaches too due to this.

(W 24)

Service providers and women talked about the emotional and psychological effects of IPV such as anxiety, depression, suicidal thoughts and attempts, and psychosomatic manifestations of psychological effects such as wheezing, fainting episodes, and abdominal pain, etc. Both groups also talked about suicidal thoughts and attempts. Women considered taking not only their own lives, but also those of their children because they were overwhelmed by the IPV. One woman said she acted on these suicidal thoughts: “*I drank poison and gave my children too (W 16)*.*”* Another said: *“One day I drank poison*, *some kind of seeds*. *Another day I drank kerosene oil too (W 23)*.*”*

Service providers talked about women they had met in hospital:

Usually the women are not able to express their feelings. One patient came to me with wheezing episodes and she has been on IV lines, nebulized many times. The other one was having fainting episodes. They can’t or won’t express what is happening to them.

(SP 06)

Due to the overwhelming nature of what is happening to them they decide to commit suicide, sometimes with their children. This happens in the form of burning themselves or jumping in front of the trains.

(SP 10)

### Better services, same outcomes

In general, service providers believed that over the years, women were increasingly disclosing their experiences of IPV and seeking help from agencies providing related services. Some community leaders felt that this was a result of their own work and the work of others which had raised awareness, changed the common perception that IPV was a private matter, and increased the availability of services for women.

Earlier women used to think it [IPV] was a private matter and they did not want to tell outsiders about it because of worry about their reputation. Now, in the villages, we can see that they have the legal and counselling supports. Based on the past 19 years of my experience, I can safely say that women are now disclosing their problems.

(SP 08)

Because of the awareness programs, men are scared that women will go to GBV desk [Gender Based Violence desk].

(SP 02)

The reported cases are higher in the resettled villages. Because of the work we have done in the Eastern Province, the women are getting more confident to report. We can see more cases being reported.

(SP 15)

However, service providers also felt that women’s vulnerability to IPV or their ability to leave abusive relationships had not changed. In fact, they felt that their ability to help women was limited, because both men and women in this region believed in the men’s right to control their wives. Although IPV was a common problem and affected many women, they said only a few would seek services.

50% of women in Battilana district is facing this problem. For a month, we will get 20 to 25 cases.

(SP 03)

Even if we advise and do counselling, it is not worthwhile because the aggressor thinks that he is doing what is right, and beating is good and he can change the wife.

(SP 13)

Usually the women are not able to express their feelings so they don’t share the feeling [what is happening to them]. They don’t express.

(SP 06)

But wives think that everything should happen according to the husband’s will. This kind of mentality is there among the women over here. Our society is having an ideology that women should listen to men, it is still in our societies.

(SP 11)

Women also recognized that more service are available now, and some had tried to access help. One said: “*I got admitted to the hospital and complained about him to the police*, [*but*] *these things didn’t scare him* (*W 16*)” referring to how reporting her abuser to police did not stop the violence.

Other women said they were reluctant to seek services because they feared that this would lead to more severe or frequent violence to themselves and/or harm to their children. One woman said: “*I didn’t make any complaints because he told [me] that he would kill me and my children if I complained to anyone (W 24)*.*”*

Some women who had contacted the police said they received no help because the men had more power over the women or they had connections to militant groups, so the police did not want to intervene. One health care provider said: “*The problem is this man joined the militant groups*, *so [Police] don’t want to find him*, *arrest him*, *or catch him*. (SP 15).”

Another woman said: “*They [police] didn’t help me because he [the husband] told them that I had connections with a terrorist group (W 22)*.*”*

Some women described how they had lived with the abusive partner for many years. Some escaped from the violence or sought help from family, but these were generally temporary measures to avoid the abuse, rather than to permanently leave the abuser.

I never stay if he comes home after drinking. I take them [the children] away. When I know that he is drunk I go out in the evenings, taking my children with me.

(W 22)

His brother was living next door, he used to come and help me. After my husband scolded them in inappropriate manner they also stopped coming and becoming involved in our problems.

(W 16)

The few women who left their abusive husbands talked about being able to go abroad to earn money and become independent. However, this process was difficult and lengthy and was not an option for most the women.

One woman was able to leave her abusive husband after working abroad and saving some money so that she could be financially independent: “*At the end I went abroad*, *it was not easy at that time to go abroad you know*. *I managed to get some money from someone*, *and I promised that I will return it after I get my first salary*. *They also agreed for that*. *They arranged everything and sent me over there*. *After I went there I started to send the money to them*. *I banked some money in the fixed deposit*, *from the interest we are living (W 18)*.*”*

Another woman said her husband stopped abusing her after she became more economically independent: “*I went to Singapore leaving my children with my mother*. *I Worked there for 4 years and arranged marriages for my 2 children*. *I built a small shop*, *I brought chickens and sold*. *I also used to stitch [clothes]*. *For the last one year*, *he stopped hitting me (W 16)*.*”*

## Discussion

Militarized violence and gender-based violence against women have been reported from the war-affected areas of Sri Lanka [31]. However, less is known about IPV in these settings. The service providers and women participating spoke about the varied forms of IPV experienced by women in the Eastern Province of Sri Lanka.

The results revealed similarities with the IPV experiences of women in other parts of Sri Lanka in terms of severity and the different forms of IPV [15,22,23] but they also revealed vulnerabilities for Tamil and Muslim ethnic-minority women compared to Sinhalese women in this post-war context. This was partly linked to the prolonged and continued community experiences of violence and acceptance of violence as a way of dealing with conflict, as well as widespread feelings of insecurity and fear about community violence among the Muslim and Tamil communities [32]. Community violence and acceptance of violence as the norm has been linked to IPV risk in many different contexts. In the context of the current study, we also observed a considerable presence and the involvement of military personnel in civil administrative work in this region, even after the war. We also sensed fear and anxiety among the community in general, and among women in particular, which could be related to the numerous incidents of sexual violence and rape in the region [13]. Within this context, women could be seen as needing more protection than before, because they could be at increased risk of rape and other forms of gender-based violence while in public. This may explain women’s continued—and perhaps increased—dependency on their husbands, and also their increased vulnerability to IPV [33]. This could also be understood as women exercising agency in negotiating their safety by aligning themselves with the lesser of two evils, the (abusive) men from their own community (compared to the military) [34]. Another consequence of the need to protect women in this region is underage marriages. Child marriage is a known consequence of conflict: parents may give their daughters in marriage to prevent them from being recruited by the militant group [35] or encountering various other forms of harm.

With death, disappearances, disability and loss of traditional sources of livelihood (such as fishing) affecting many men, women in war-affected areas of Sri Lanka have taken on bread-winner and head of household roles, and have become more active in their communities. When there is a disruption of established social relationships and roles in war and post-war contexts, men are known to use violence as means of re-exerting control and power to maintain roles that are consistent with social norms [11]. This appears to be the case in the current region of study, where men have been removed from valued and familiar roles because of the changing economic, political and social context. The service providers we interviewed said that men used violence to ‘save’ women who had stepped outside ‘acceptable boundaries’ of freedom for women. Previous research has found that both men and women living in societies marked by traditional gender norms may believe that husbands have the right to use violence against their wives in order to correct their behavior [36].

Because gender inequality is shaped by its intersection with other systems of power, the dynamics of IPV with a society must be understood not only within the context of different forms of oppression experienced by women living with IPV, but also within the context of their (struggle for) resistance [37]. Women in certain post-armed conflict/war societies have been able to overcome some gender-based oppression and to negotiate changes in gender roles that are favourable for them. For example, in post-conflict regions in northern Uganda [38], Sierra Leone, and Liberia [8], when men’s status was diminished due to lack of employment and loss of their primary or sole bread-winner status, women were able to improve their economic, political, and social status. In post-conflict Sierra Leone, women gained more financial independence and decision-making ability when long-standing cultural norms changed in favour of women. However, at the beginning of this process, they became more vulnerable to IPV as men tended to use violence to reinstate their status, a situation similar to that in the current region of study. While women’s economic independence or solvency may increase their options for dealing with IPV, it can also increase their risk of IPV when abusive partners are threatened by the woman’s new improved social status [39]. Most of the interviewees in our study felt that increased access to work outside the home and somewhat better financial freedom made them more vulnerable to IPV; only a few women had found economic independence and recourse from abuse as a result of this change.

Service providers and women referred to significant psychological effects of IPV, including heightened risks of self-harm and suicide. The mental health consequences of IPV have generally not been documented in Sri Lanka, and even when reported the severity and extent of these issues has not been clarified. In post-war Eastern Province, most of the population is likely to be dealing with war trauma [10,21,40], and the link between IPV and mental health and wellbeing may be masked within this context of complex psychosocial issues [41,42]. The cumulative effects of IPV and other forms of violence on the mental health of women needs to be considered, particularly in this context.

Across Sri Lanka, very few women disclose IPV, and of those who do, most seek help from close family networks rather than from formal agencies [22,23,26]. Women interviewed for our study responded to IPV in a similar manner, by either accepting it or by seeking help from close family. Although the service providers we interviewed talked about improved IPV-related services in the Eastern Province, according to the women, only a few said they had accessed services. Most were afraid to disclose IPV because their husbands had threatened them with more violence. In the context of community violence, men having access to weapons, and some being directly or indirectly connected to militant groups, women were fearful of these threats. Additionally, the stigma associated with divorce or separation adversely affects women in all parts of Sri Lanka [22–26]. Together, all of these factors prevented women from seeking services.

Some structural barriers could add another level of complexity for women dealing with IPV in this region. People in the Eastern Province have been displaced numerous times, and many have lost documents (e.g., national identity cards and marriage certificates) [11] that they need to access government services. Also, at the time of this study, the civil service, justice system, and health and social services were not yet fully re-established in the Eastern Province [12]. Finally, the existing services had extensive involvement of the Sinhalese military in their administration [13]. Tamil and Muslim women are likely to feel afraid to seek services when there is a prominent Sinhalese military presence in towns and near services. This might have been an important reason why the women we interviewed did not seek services, but none explicitly referred to it. The end of the war may have been too recent to allow them to discuss their concerns openly.

### Limitations

This study had some limitations that may have affected the results. While we have framed our findings primarily in terms of gender inequities and the post-war context, we note that there is considerable congruence between our findings and research focussed on IPV among women living in rural setting, particularly the role of traditional gender roles and the power of these accepted gender norms in shaping women’s experiences and options, in light of the oppressive conditions they face and the limited supports and services available. Although this would have been a useful additional consideration in this study, the small number of participants interviewed does not allow us to go beyond the present analysis.

## Conclusions

Our goal was to generate contextual and locally-relevant knowledge about the unexplored phenomenon of IPV in the post-war context of the Eastern Province of Sri Lanka, examining how it is produced and how women respond to it. We found out that changing gender roles colluded with patriarchal ideologies and intersected with other conditions to increase women’s vulnerability to IPV. The ethnic dimensions of the civil war, and the continuing ethnic tensions post-war, made the situation worse for Tamil and Muslim women creating conditions that are likely to keep them entrapped in abusive relationships. Although there are more opportunities for women to access work and gain economic independence from men post-war, the positive effects on women’s lives would not be seen until the patriarchal ideologies and ethnic tensions have had chances and time to be resolved.

From a research perspective, our findings underscore the need for further study into IPV among vulnerable communities, particularly in post-war contexts as such women’s experiences may be influenced and also masked by a complex set of factors which intersect to both produce IPV and to entrap women in violence. Insights from such work could provide a more nuanced understanding of context-specific issues shaping women’s experiences of IPV and community responses to it, and ultimately, to the development of more comprehensive solutions which fit the local context.

From a practice and service perspective, the study draws attention to the complex links between war-related trauma, psychosocial issues, and the psychological consequences of IPV. Further in-depth study is needed about the ways in which the community is responding to post-war changes including the control of women, the women’s need for protection, and marriage of young girls. This knowledge is relevant for healthcare professionals, social workers, and community leaders who work with and provide health, social, and IPV-related services to war-affected communities.

From a policy perspective, complex solutions are needed to provide accessible and effective safety and health options for women in post-war contexts. It requires policies aimed at reconciliation and rehabilitation to address unresolved ethnic tensions and to build confidence and harmony within highly polarized post-war societies.
